# QTL variations for growth-related traits in eight distinct families of common carp (*Cyprinus carpio*)

**DOI:** 10.1186/s12863-016-0370-9

**Published:** 2016-05-05

**Authors:** Weihua Lv, Xianhu Zheng, Youyi Kuang, Dingchen Cao, Yunqin Yan, Xiaowen Sun

**Affiliations:** College of Life Science, Northeast Agricultural University, Harbin, 150030 China; Heilongjiang River Fisheries Research Institute, Chinese Academy of Fishery Sciences, Harbin, 150070 China

**Keywords:** *Cyprinus carpio*, Growth-related traits, Linkage maps, QTL, Multiple families

## Abstract

**Background:**

Comparing QTL analyses of multiple pair-mating families can provide a better understanding of important allelic variations and distributions. However, most QTL mapping studies in common carp have been based on analyses of individual families. In order to improve our understanding of heredity and variation of QTLs in different families and identify important QTLs, we performed QTL analysis of growth-related traits in multiple segregating families.

**Results:**

We completed a genome scan for QTLs that affect body weight (BW), total length (TL), and body thickness (BT) of 522 individuals from eight full-sib families using 250 microsatellites evenly distributed across 50 chromosomes. Sib-pair and half-sib model mapping identified 165 QTLs on 30 linkage groups. Among them, 10 (genome-wide *P* <0.01 or *P* < 0.05) and 28 (chromosome-wide *P* < 0.01) QTLs exhibited significant evidence of linkage, while the remaining 127 exhibited a suggestive effect on the above three traits at a chromosome-wide (*P* < 0.05) level. Multiple QTLs obtained from different families affect BW, TL, and BT and locate at close or identical positions. It suggests that same genetic factors may control variability in these traits. Furthermore, the results of the comparative QTL analysis of multiple families showed that one QTL was common in four of the eight families, nine QTLs were detected in three of the eight families, and 26 QTLs were found common to two of the eight families. These common QTLs are valuable candidates in marker-assisted selection.

**Conclusion:**

A large number of QTLs were detected in the common carp genome and associated with growth-related traits. Some of the QTLs of different growth-related traits were identified at similar chromosomal regions, suggesting a role for pleiotropy and/or tight linkage and demonstrating a common genetic basis of growth trait variations. The results have set up an example for comparing QTLs in common carp and provided insights into variations in the identified QTLs affecting body growth. Discovery of these common QTLs between families and growth-related traits represents an important step towards understanding of quantitative genetic variation in common carp.

**Electronic supplementary material:**

The online version of this article (doi:10.1186/s12863-016-0370-9) contains supplementary material, which is available to authorized users.

## Background

The common carp (*Cyprinus carpio*) is one of the most widespread freshwater teleost species in the world. It has been domesticated as an important food fish in over 100 countries worldwide with global production exceeding 3.79 million tons in 2012, according to the Food and Agriculture Organization [1]. This globally important aquaculture species is used as a model in many research areas, e.g., ecology, physiology, and evolution. Significant progress has been made in common carp genetics and genome research over the last decade. The current genomic resources available for common carp include polymorphic genetic markers [2, 3], genetic linkage maps [4–6], QTLs [7–10], cDNA microarrays [11], bacterial artificial chromosome libraries [12, 13], and physical maps [14]. Furthermore, A draft sequence of the common carp genome has been assembled [15], which provides reference sequences for genomic and comparative genomic studies of all common carp strains and other Cyprinidae species. All of these resources, which are available online (http://www.carpbase.org), lay the foundation for future studies on the genetic mechanisms of economic traits in common carp and related species.

Growth is an economically important trait in the fish farming industry because it is directly related to fish production. An improvement in growth rate increases benefits for aquaculture companies because it decreases the raising time at farm facilities, leading to lower costs and higher harvests [16]. Growth-related traits such as body weight, total length, standard length, body depth, and body thickness are quantitative traits influenced by both environmental factors and multiple genes with relatively small effects according to the infinitesimal model [17]. Traditional selective breeding techniques are valuable in achieving improved fish growth [18], but the genetic gain can be increased far faster with MAS [19].

More recently, there has been an increasing number of studies that identify QTLs for growth-related traits in food fish species [20–22]. Most have been carried out in salmonids (e.g., Atlantic salmon [23, 24], rainbow trout [25], and coho salmon [26]), tilapia [27], sea bass [28, 29], and turbot [30, 31] as well as in common carp, involving backcross [32] and F_1_ [9, 33, 34] populations. However, all of these common carp QTL studies were limited to a single segregating family and small population size (~46–190 samples). In our previous study, through comparing the distribution and variation of QTLs in four half-sib families, we found that the major QTLs for growth-related traits were not fixed either between or among families. The study also revealed that both major and minor genes differ in their genetic performance. Therefore, we concluded that the major genes are undergoing change and remain unfixed in these families [35]. Further investigation is needed to identify shared and overlapping QTLs in either populations or families to understand quantitative genetic variation.

In the present work, we conducted a genome scan for QTLs that affect body weight, total length, and body thickness in eight full-sib families containing 522 individuals from a breeding population. The objectives of this current study were: 1) to locate QTLs on linkage groups, 2) to identify which QTLs are either common or specific to all families, 3) to detect common and overlapping QTLs for the three growth-related traits, and 4) to explore the genetic architecture of growth-related traits.

## Results

### Phenotypic variation

Analysis of the raw phenotypic data in the eight full-sib families revealed that all traits showed substantial levels of phenotypic variation. The average values and associated dispersion measures for BW, TL and BT are summarized in Table 1. The eight families were F234, F275, F4039, F171, F217, F373, F336 and F259, which contained 45–107 progeny. The mean value for BW, TL and BT within each family ranged from 475.9 to 722.9, from 29.8 to 36.1 and from 45.0 to 53.8, respectively. The high dispersal of means across the families resulted from the family effect caused by all of the fish being fed in the same pond. Heritabilities and correlations for the three growth-related traits are shown in Table S1 (Additional file 1). Moderate heritabilities were observed for the three traits, 0.23 for BW, 0.35 for SL and 0.25 for BT. Highly and significantly phenotypic correlations (0.83–0.92) and genetic correlations (0.93–0.98) were observed among the three traits.

**Table 1:** Phenotypic values of growth-related traits in the eight common carp full-sib families

Parental pair	Family name	N	Body weight (g)		Total length (cm)		Body thickness (mm)	
			Mean	SD	Mean	SD	Mean	SD
♀23 × ♂4	F234	107	612.4	159.1	34.0	3.0	51.1	5.4
♀27 × ♂5	F275	70	603.2	181.6	34.1	4.7	49.7	6.9
♀40 × ♂39	F4039	70	527.2	204.8	31.2	4.8	46.9	6.7
♀17 × ♂1	F171	69	599.0	181.3	33.5	3.6	49.9	5.1
♀21 × ♂7	F217	65	475.9	186.7	29.8	4.0	45.0	6.1
♀37 × ♂3	F373	50	589.8	167.5	33.4	2.8	48.0	5.9
♀33 × ♂6	F336	46	555.6	165.9	31.9	3.2	48.1	5.8
♀25 × ♂9	F259	45	722.9	236.1	36.1	3.9	53.8	7.1

*N* number of individuals, *SD* standard deviation

### QTLs for growth-related traits

**Fig. 1:**
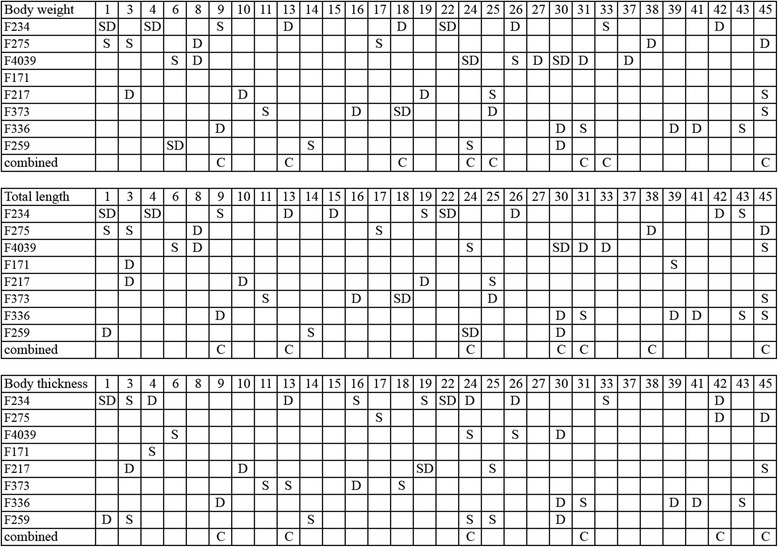
Comparison of QTLs detected among the 30 chromosomes in the eight common carp families. S and D represent the QTL based on sire- and dam half-sib analysis, respectively; combined indicates the QTL based on sib-pair analysis

QTLs that exceeded the suggestive or significant linkage threshold are revealed in Tables 2, 3, 4 and 5 and Additional file 1: Tables S2–S4. We found evidence for QTLs affecting BW on 29 LGs, TL on 28 LGs, and BT on 25 LGs. Thus, QTLs affecting all traits were found on 30 of the 50 common carp chromosomes covered by the linkage maps (Fig. 1). A total of 165 QTLs were detected across all traits by the whole genome scan. Out of the 165 QTLs, ten (six at genome-wide *P* <0.01 and four at genome-wide *P* < 0.05) and 28 (chromosome-wide *P* < 0.01) exhibited significant evidence of linkage, while the remaining 127 exhibited a suggestive effect on the three growth-related traits at the chromosome-wide (*P* < 0.05) level.

**Table 2:** Suggestive (*P* < 0.05) and significant (*P* < 0.01) QTLs from the sib-pair genome scan

Trait	LG	Position (cM)	*F*-ratio	*F*-statistic threshold				V_gQTL_ (SE)	CI	Nearest marker
				Chromosome-wide		Genome-wide				
				0.05	0.01	0.05	0.01			
Body weight	9	89	17.62	16.61	31.66			433.76(11.46)	87–89	CAFS2311
	13	8	10.86	8.49	14.76			421.79(10.80)	0–8	HLJ3991
	18	55	6.02	5.75	9.48			408.26(8.90)	36–60	HLJE299
	**24**	53	**36.05**	8.67	15.37	32.75	36.01	435.11(8.77)	34–56	HLJE511
	25	67	9.75	8.61	15.66			414.14(9.09)	51–74	HLJ3952
	31	58	10.94	8.05	14.20			417.94(9.71)	0–72	HLJ2149
	33	14	5.57	5.57	11.19			404.89(7.94)	13–36	HLJ2143
	45	46	16.87	7.26	13.10			419.03(8.45)	35–131	HLJ3597
Total length	9	89	28.18	18.01	35.81			32.33(0.88)	88–89	CAFS2311
	13	8	10.52	8.83	15.25			30.46(0.83)	0–8	HLJ3991
	**24**	54	**25.43**	8.47	15.67	25.35	29.81	30.88(0.66)	34–56	HLJE511
	30	117	17.57	16.93	31.09			36.35(2.03)	76–127	CAFS873
	31	65	9.77	8.09	14.13			30.45(0.86)	0–77	HLJ2149
	38	46	10.68	10.45	17.37			30.96(0.97)	45–56	HLJ3291
	**45**	46	**20.56**	7.41	13.52	20.07	29.05	30.52(0.65)	36–131	HLJ3597
Body thickness	9	89	17.23	17.19	35.01			1.82(0.49)	76–89	CAFS2311
	13	8	16.83	8.68	15.57			1.80(0.05)	0–10	HLJ3991
	**24**	56	**28.91**	8.59	15.39	26.76	31.70	1.79(0.04)	34–56	HLJE511
	31	70	9.65	8.59	15.42			1.77(0.05)	0–78	HLJ3848
	42	0	7.19	6.35	12.29			1.73(0.04)	0–3	CAFS1757
	45	46	11.03	7.15	12.11			1.73(0.04)	30–131	HLJ3597

LG, linkage group; Position (cM) on the LG where the maximum F-statistic value was obtained; V_gQTL_, the QTL variance; SE, standard error for the QTL variance; CI, 95 % confidence interval; LGs and *F*-ratios in bold are significant at genome-wide level

**Table 3:** Significant (*P* < 0.01) QTLs for body weight in eight common carp families from half-sib genome scans

Family	Source	LG	Position (cM)	*F*-ratio	*F*-statistic threshold				Estimate (SE)	ABS (t)	PVE	CI	Nearest marker
					Chromosome-wide		Genome-wide						
					0.05	0.01	0.05	0.01					
F234	Sire	4	53	12.98	5.96	10.26			−10.60(2.94)	3.60	27.2	44–53	HLJ2952
F234	Dam	**4**	53	**22.40**	5.39	8.89	15.81	18.11	−13.49(2.85)	4.73		44–53	HLJ2952
F373	Sire	11	5	14.13	6.33	10.25			−11.57(3.07)	3.76	42.2	0–49	CAFS2305
F373	Sire	18	60	12.64	5.32	8.14			11.32(3.18)	3.55	17.6	36–60	HLJE299
F4039	Sire	24	34	10.85	3.92	6.55			−13.12(3.98)	3.29	25.2	12–56	HLJ3754
F259	Sire	**24**	44	**40.00**	5.57	8.99	16.37	23.26	−25.42(4.02)	6.32	48.7	23–56	HLJ3988
F234	Dam	26	33	9.88	5.88	8.63			7.78(2.47)	3.14	15.6	0–58.5	CAFS2321
F4039	Dam	**30**	22	**14.35**	6.54	10.13	14.31	18.63	23.37(6.17)	3.79	35	6–123	--

LG, linkage group; Source indicates which parent segregated for the QTL; Position (cM) on the LG where the maximum F-statistic value was obtained; ABS(t), Absolute T value; PVE is the proportion of phenotypic variation explained by the QTL estimated using both the sire and dam analyses; CI, 95 % confidence interval; LGs and *F*-ratios in bold are significant at the genome-wide level

**Table 4:** Significant (*P* < 0.01) QTLs for total length in eight common carp families from half-sib genome scans

Family	Source	LG	Position (cM)	*F*-ratio	*F*-statistic threshold				Estimate (SE)	ABS (t)	PVE	CI	Nearest marker
					Chromosome-wide		Genome-wide						
					0.05	0.01	0.05	0.01					
F259	Dam	1	34	10.79	6.31	9.79			−4.86(1.47)	3.29	37	6–32	HLJ3473
F217	Dam	3	9	9.08	5.15	8.80			3.48(1.15)	3.01	23.4	2–51	HLJ3461
F234	Sire	4	53	8.81	5.55	8.39			−2.19(0.74)	2.97	27	10–53	HLJ2952
F234	Dam	**4**	50	**20.54**	5.48	8.63	14.43	18.44	−3.33(0.73)	4.53		41–53	HLJ2952
F4039	Dam	8	12	9.34	5.11	8.14			−3.48(1.14)	3.06	21.8	0–41	HLJ3865
F217	Dam	10	4	14.00	5.23	8.49			−6.18(1.65)	3.74	35.2	4–40	HLJ2593
F234	Dam	13	62	10.64	5.35	8.55			1.99(0.61)	3.26	16.8	0–62	HLJ2571
F259	Sire	**24**	44	**33.67**	5.29	8.97	18.78	32.12	−5.43(0.94)	5.80	43.2	28–56	HLJ3988
F4039	Dam	30	20	11.69	6.62	10.1			5.91(1.73)	3.42	27.2	0–127	--
F4039	Dam	37	18	11.29	4.81	9.93			3.91(1.16)	3.36	26.2	8–21	HLJ3542
F275	Dam	38	46	8.74	5.37	7.62			−3.38(1.14)	2.96	21.2	0–46	HLJ3291
F336	Dam	41	14	11.56	5.73	9.71			−3.62(1.06)	3.40	38	0–22	CAFS2332
F336	Sire	43	15	9.37	5.64	8.53			3.18(1.04)	3.06	31.4	5–42	HLJ360
F275	Dam	45	50	11.75	7.16	10.58			4.13(1.21)	3.43	28.4	33–131	HLJ3597

LG, linkage group; Source indicates which parent segregated for the QTL; Position (cM) on the LG where the maximum F-statistic value was obtained; ABS(t), Absolute T value; PVE is the proportion of phenotypic variation explained by the QTL estimated using both the sire and dam analyses; CI, 95 % confidence interval; LGs and *F*-ratios in bold are significant at the genome-wide level

**Table 5:** Significant (*P* < 0.01) QTLs for body thickness in eight common carp families from half-sib genome scans

Family	Source	LG	Position (cM)	*F*-ratio	F-statistic threshold				Estimate (SE)	ABS (t)	PVE	CI	Nearest marker
					Chromosome-wide		Genome-wide						
					0.05	0.01	0.05	0.01					
F234	Dam	4	52	14.33	5.56	9.31			−0.75(0.19)	3.78	22.4	40–53	HLJ2952
F336	Dam	9	48	9.24	5.46	8.06			−1.51(0.49)	3.04	31	13–89	CAFS1291
F373	Sire	13	0	10.66	6.51	10.64			−3.51(1.07)	3.27	33	0–60	HLJ2650
F234	Dam	13	0	8.82	5.67	7.57			0.48(0.16)	2.97	13.8	0–62	HLJ2650
F234	Sire	22	17	9.52	4.93	7.67			−0.73(0.23)	3.09	14.8	0–59	HLJ2491
F234	Dam	22	17	9.52	5.03	8.38			−0.73(0.23)	3.09		0–58	HLJ2491
F259	Sire	**24**	40	**28.06**	5.72	9.75	18.91	25.98	−1.56(0.29)	5.29	38.6	24–56	HLJ3988
F4039	Sire	24	56	14.01	4.47	7.96			−0.85(0.22)	3.74	32.2	2–56	HLJE511
F4039	Dam	30	22	15.26	6.24	10.28			1.38(0.35)	3.91	34.6	7–127	--
F336	Dam	41	16	11.31	5.91	9.07			−0.99(0.29)	3.36	37.2	0–24	CAFS2332

LG, linkage group; Source indicates which parent segregated for the QTL; Position (cM) on the LG where the maximum F-statistic value was obtained; ABS(t), Absolute T value; PVE is the proportion of phenotypic variation explained by the QTL estimated using both the sire and dam analyses; CI, 95 % confidence interval; LG and *F*-ratio in bold is significant at the genome-wide level

For BW, the sib-pair analysis using shared markers among all eight families, identified two significant QTLs on LG24 and LG45, and six LGs containing suggestive QTLs for this trait (Table 2). A single QTL, on LG24 at 53 cM reached the genome-wide (*P* < 0.01) significance threshold. Additionally, the half-sib analysis revealed eight significant QTLs on six LGs (4, 11, 18, 24, 26, and 30), whereas 42 suggestive QTLs were detected on 29 LGs (Table 3, Additional file 1: Table S2). Three out of the eight significant QTLs surpassed the genome-wide significance threshold; of these, two QTLs at the genome-wide (*P* < 0.01) level located on LG4 in F234 segregated from the dam and LG24 in F259 segregated from the sire, and one QTL located on LG30 in F4039 that segregated from the dam reached the genome-wide (*P* < 0.05) significance threshold, accounting for 27.2–48.7 % of PVE. Seven QTLs were found to segregate from both sire- and dam-based analysis, these were located on LG1, LG4, and LG22 in F234, LG24, and LG30 in F4039, LG18 in F373, and LG6 in F259. The QTL found on LG30 was common to F336, F259, and F4039, and although they were located close together on the chromosome, the QTL in F336 and F259 segregated from the dam, whereas it segregated from both parents in F4039. The QTL on LG45 was common to F275, F217, and F373, and although they were positioned close together in the LG, the QTL in F217 and F373 segregated from the sire, whereas it segregated from the dam in F275.

Sib-pair QTL analysis for TL based on shared markers for all families identified significant QTLs (genome-wide *P* < 0.05) on two LGs (24 and 45) and suggestive QTLs (chromosome-wide *P* < 0.05) on five LGs (9, 13, 30, 31, and 38) (Table 2). The half-sib analysis detected 13 LGs (1, 3, 4, 8, 10, 13, 24, 30, 37, 38, 41, 43, and 45) containing 14 significant and 37 suggestive QTLs in 23 LGs (Table 4, Additional file 1: Table S3). Most of the significant QTLs were detected in only one of the families. Two significant QTLs, on LG4 in F234 that segregated from the dam and LG24 in F259 segregated from the sire reached the genome-wide (*P* < 0.01) level of significance, accounting for 27 % and 43.2 % of PVE, respectively. The QTL on LG45 was detected in four families (F275, F336, F373, and F4039), but only one of them that segregated from the dam in F275 was significant (chromosome-wide *P* < 0.01) and contributed 28.4 % of PVE. The QTL on LG1 was common to F234, F259, and F275, and although they were close to 37 cM on the LG, the QTL segregated from the dam in F259 and from the sire in F275, whereas it segregated from both parents in F234. Three families (F259, F336, and F4039) had QTLs segregating from the dam on LG30, and although they were close together on the chromosome, only one significant QTL (chromosome-wide *P* < 0.01) was found in F4039.

For BT, the sib-pair analysis identified significant QTLs on two LGs (13 and 24) and suggestive QTLs on four LGs (9, 31, 42, and 45) (Table 2). Only the QTL found on LG24 reached the genome-wide significance threshold (*P* < 0.05). This QTL was not only located on the same position at 54 cM on LG24, but also reached a genome-wide level of significance (*P* < 0.05) in all three traits (BW, TL, and BT). In the half-sib analysis, a total of 43 QTLs dispersed over 25 LGs were associated with BT, and from these, ten QTLs in 7 LGs (4, 9, 13, 22, 24, 30, and 44) were significant (Table 5) and 33 suggestive QTLs were found on 23 LGs (Additional file 1: Table S4). Only one QTL found on LG24 reached the genome-wide significance threshold (*P* < 0.01), and contributed 38.6 % of PVE. At two locations, a significant QTL was found at the same position: 17 cM on LG22 in F234 and 0 cM on LG13 in families F373 and F234. The PVE by the individual QTL varied from 13.8 to 33 %. QTLs segregating from the dams in F259, F336, and F4039 were detected at similar positions on LG30, but only one of them reached the chromosome-wide level of significance (*P* < 0.01) and accounted for 34.6 % of PVE in F4039. The QTL on LG45 was common to F217, F259, and F275, and all of them were suggestive, the QTL in F217 and F259 segregated from the sire whereas it segregated from both parents in F275. QTLs segregating from the sires in F217 and F259 were detected at similar positions on LG25.

### Comparison of growth-related trait QTLs

**Fig. 2:**
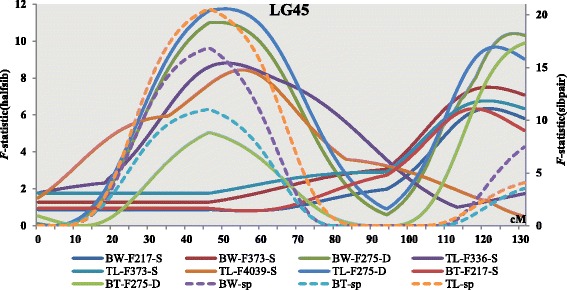
*F*-distributions for QTLs on common carp LG45 affecting body weight, total length and body thickness. The *F*-distributions for the half-sib analyses are plotted against the primary y-axis, and the *F*-distribution for the sib-pair analysis is plotted against the secondary y-axis. The x-axis indicates the relative positions of the markers on the linkage map (cM). F217, F373, F275, F336, and F4039 are the family names, S and D represent sire- and dam-based half-sib analysis. sp indicates sib-pair analysis

Although most QTLs were either detected in just one family or at different positions on the map, 36 (24.5 %) QTLs common to all families were observed in the half-sib analysis (Fig. 1). For a single trait, one QTL common to four families, nine common to three families and 26 common to two families were identified. However, no QTL was common to more than five families. The QTL for TL located on LG45 was common to four families (F275, F336, F373, and F4039); it segregated from the dam in F275 and from the sire in F336, F373 and F4039. Similarly, the QTL for BW on LG45 was detected in three families (F275, F217, and F373) and segregated from the dam in F275 and from the sire in F217 and F373. The QTL for TL on LG45 was detected in two families (F275 and F217); it also segregated from the dam in F275 and from the sire in F217. However, five QTLs were located close to 50 cM and four QTLs harbored near120 cM on LG45 (Fig. 2). Meanwhile, we found many QTLs (16 QTLs for BW, 14 QTLs for TL and 13 QTLs for BT) that were detected only in one family. There might be many reasons for the presence of family-specific QTLs, including family-specific marker regression coefficients [36], fixation of both the QTL and markers in some families, and the linkage phase between marker and QTL alleles might vary between families.

As shown in Fig. 1, the different traits have sharing QTLs in multiple families. Approximately two LGs (3 and 45) with overlapping QTL regions associated with all traits across five families were identified; four LGs (1, 24, 25, and 30) with common QTL regions related to all traits were identified in three families; five LGs (4, 16, 18, 39, and 42) with common QTLs affecting all traits in two families; and 11 LGs with common QTLs affecting all traits in a single family. For example, QTLs influencing all traits in F373 were detected at similar positions on LG18, which segregated from the sire for BT and from both parents for BW and TL. Additionally, another QTL for BW also was found to have common regions on LG18 that segregated from the dam in F234. The QTL associated with all traits had common regions on LG1, which segregated from both parents in F234, from the sire in F275, and from the dam in F259.

### Candidate gene markers for growth-related traits

The sequences of 57 loci that appeared next to QTL peaks were BLAST searched against the common carp genome sequences. The results presented that all of the sequences had significant hits on the common carp genome. Further analysis indicated that 20 sequences had very high similarity to common carp annotation genes (Additional file 1: Tables S2–4). One gene (*farsb*) was associated with growth, three with lipid metabolism (*apobl*, *adiporla*, and *lpcat1*), three with muscle development (*mybcp2a*, *mbn12*, and *foxk2*), and five with ionic transport and physiology (*pcdh2ac*, *clcn3*, *vps13b*, *stim1a*, and *casq2*).

## Discussion

### Methodological comparison

In our study, the datasets were analyzed by two different methods. First, analyses were performed using a sib-pair model to make use of the full-sib pedigree structure and the genotypes of the parents. Second, we carried out half-sib analyses to identify segregating alleles in sires and dams with a QTL. This two-stage analytical method was used by Gutierrez et al. in their QTL investigation in Atlantic salmon [23]. Together with the data from the eight families into one analysis and each family analysis separately, we can determine which family has a specific QTL. In the sib-pair analysis, the QTL affecting all three traits on LG24 was highly significant, exceeding the 0.01 and 0.05 significance thresholds at the genome-wide level. In comparison, the half-sib analysis detected the same QTL on LG24, which influences all traits shared by three families (F234, F259, and F4039), evidence for this QTL also reached the 0.01 genome-wide significance level in F259 based on sire analysis, and surpassed either 0.01 or 0.05 chromosome-wide significance level in F234, F259, and F4039 based on sire and dam analysis. Similarly, the QTL affecting standard length (SL) identified on LG45 was detected in both the sib-pair and half-sib analyses, exceeding the 0.01 genome-wide threshold in the sib-pair analysis, 0.01 chromosome-wide threshold in F275 based on dam half-sib analysis, and 0.05 chromosome-wide in F336, F373, and F4039 based on sire half-sib analysis. Therefore, there was a better consistency between the sib-pair and half-sib analyses in their ability to identify and position this QTL.

### Relationships between the QTLs identified in this study and those in the literature

Growth-related traits (e.g., body weight and length) constitute the main purpose of genetic breeding projects in aquaculture. Earlier studies on common carp have confirmed a heritable basis in these traits [37], and some QTLs affecting growth-related traits have been reported [32–35, 38, 39]. For example, Laghari et al. [32] performed analysis looking for BW, TL, and condition factor (K) based on a backcross common carp family, they detected 12 QTLs associated with BW and TL on ten LGs. Laghari et al. [33] based their BW QTL analysis on phenotypic data obtained from 3-month points ranging from 10 to 12 months of culture, and they detected seven QTLs on three LGs. Zhang et al. [34] described the identification of SL QTLs using an F_1_ common carp family, they located four QTLs affecting SL on four LGs. However, previous QTL studies related to growth traits in common carp have generally been conducted on a single population. The ability to detect QTLs is often limited, because only the QTLs that segregate in either one or both parents can be detected [40]. The QTLs identified may not be representative of the genetic architecture of common carp growth. Therefore, the QTLs detected should be validated in other common carp families before they are used in genetic breeding. Comparing our QTL results with previously detected QTLs in populations with different genetic backgrounds, the QTL on LG22 reached 0.01 chromosome-wide significance for BT, which segregated from both parents in F234, this is similar to the QTL affecting SL found in a full-sib family [38]. Another significant QTL (chromosome-wide *P* < 0.01) for TL on LG13 in F234, also had an overlapping QTL region with that associated with SL reported by Zheng et al. [38]. Additionally, one QTL for BW on LG30 in F259 was identified as having a shared region with the QTL for body height (qBH30) on this chromosome in an F_1_ population [39]. Other than these examples, there are no other reports of overlap in the literature on QTL mapping for common carp growth traits.

### Comparison of growth-related trait QTLs

Overlapping QTL regions affecting all traits were detected on 22 LGs, for example, one region on LG3, a chromosomal region was associated with three traits among five families. Overlapping QTL regions suggest the possibility that common genetic factors govern all growth traits, which is similar to the results of other researches, e.g., turbot *Scophthalmus maximus* (growth-related QTL) [30]. Overlapping QTL regions among multiple traits were found on nine LGs (1, 2, 5, 6, 12, 13, and 15–17). All traits were strongly correlated (Additional file 1: Table S1), further suggesting that they may be controlled by some of the common genes. While this might be caused by the wide confidence intervals for the QTL regions detected, it is also possible that this could indicate either tight QTL linkage or pleiotropic effects on growth trait variation. For example, the significant QTL on LG24 close to marker HLJ3988, affecting all three traits, is controlled by the common gene *casq2* (*calsequestrin* 2). In some chromosome segments, QTLs identified for single traits did not overlap with QTLs for other traits, e.g., QTL for TL on LG15 and QTL for BW on LG27, suggesting that these chromosome segments contain genes that may be specifically responsible for a single trait.

### The advantages of mapping QTLs in multiple families

**Fig. 3:**
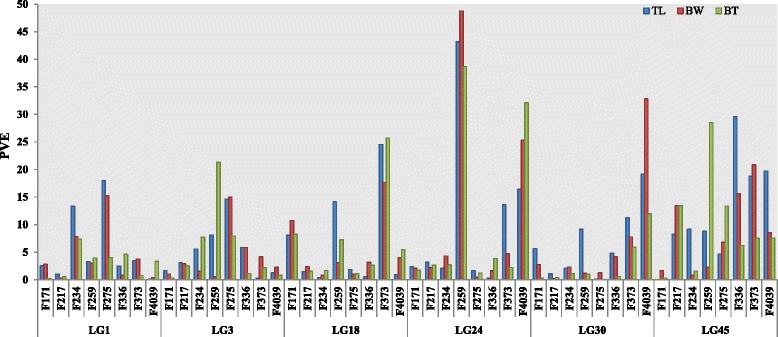
Hereditary effects on the variation of QTLs based on sire-based analysis in eight families. Abscissa indicates eight families’ name, the ordinate represents phenotypic variance explained (PVE) of QTLs. BW, body weight; TL, total length; BT, body thickness

A major limitation of QTL analysis in biparental populations is that the estimated effects are specific to that population and QTL results are often not suitable for other populations, thus restricting their use for MAS projects [41]. Therefore, mapping QTLs based on multiple segregating families allows us to identify more QTL locations, which should also allow more powerful ability to estimate the QTL effects across populations. In this study, the analysis of eight different mapping families identified more QTLs than in a single population, showed more alleles, and locate a more precise position of the QTLs that were shared by several families. Comparison of the QTL mapping results across all families showed that one QTL was common to four families, nine were detected in three families, and 26 were found in two families. The common QTLs that exist among multiple families are not only more valuable in MAS, but also probable represent relatively stable gene sites over long terms of species evolution [42]. Except for the seven common QTLs, most common QTL intervals represent existing genes, which is similar to the 60 % common QTL regions involving genes that were found in four common carp families [35]. Therefore, the authors believe that the genes that regulate traits in QTL regions are probably the basis of shared QTLs in different families. Common QTLs also showed variable phenotypic effect in terms of PVE in different families. As is shown in Fig. 3, the PVE explained by common QTLs varied. For examples, the QTL for TL on LG45 accounted for approximately 30, 20, 8.2–9.1 % of PVE in F336, F373 and F4039, and F217, F234, and F259, respectively, but less than 5 % in F171 and F275. However, only the QTL in F336, F373, and F4039 reached the significance level. The QTL for BW on LG24 contributed a high percentage, 48.7 % of the PVE in F259 and 25.2 % of PVE in F4039; however, it accounted for less than 5 % in the other six families. This might be because of the relevant loci in QTL intervals have different heterozygous states in different families, which can also be interpreted as polymorphisms at different markers in the same region contributing to genetic variation in different families. Therefore, to enhance the efficiency of MAS, it is necessary to detect QTLs that are effective across different genetic backgrounds.

### Positional candidate genes

By BLAST searching QTL loci sequences, we identified 20 markers with related genes and relevant function annotations. The functions of these genes are mainly involved in energy storage, protein synthesis, protein activity regulation, cellular components, cell differentiation, ion transport, and signal transduction, there were also some unknown functional genes. Interestingly, a particular case is that of the QTL that affects both BW and SL located on LG9 close to the gene-related marker HLJ3833, this marker is located near the *farsb* gene (*phenylalanyl-tRNA synthetase, beta subunit*), which is a candidate gene for weaning weight in Canchim beef cattle [43] as well as affecting feed efficiency in chickens [44]. The QTL associated with SL located on LG3 close to HLJ2461, which is located near *clcn3* (*chloride channel* 3), is also a candidate gene for body size in the ninespine stickleback (*Pungitius pungitius* L.) [45]. We expect that focusing on these markers that are related to traits of interest will be fruitful in investigating genes. This is because the identification of QTLs influencing different traits could increase the efficiency of MAS and enhance genetic progress.

## Conclusions

In summary, we have identified 38 significant (*P* < 0.01) and 127 suggestive (*P* < 0.05) QTLs associated with three growth traits by analyzing eight full-sib families from a breeding population. The abundant putative QTLs provide a broad view of the genetic structure of growth traits in common carp. Some of the QTLs were identified on similar chromosome segments, suggesting a role for pleiotropy and/or tight linkage in explaining the genetic basis of growth trait variation. In general, our studies lay the foundations for future comparative QTL mapping for growth-related traits and provide deeper insights into understanding and using quantitative genetic variation in common carp.

## Methods

### Fish and phenotypic measurements

The mapping population comprising eight full-sib families was used for QTL analysis of flesh fat content trait in common carp [7]. Briefly, the progeny came from 60 parents (40 female and 20 male) spawned in 2009 as part of the common carp breeding population, in Songpu Aquaculture Experimental Station, Heilongjiang River Fisheries Research Institute. Thirty full-sib families were constructed by crossing mature brooders with good performance, indicating high allelic diversity and genetic differences. After 50 days post hatching in a hatchery tank, ~2000 fish fry were randomly selected to feed in one pond for 2 years, we identified the genetic diversity and paternity relationships among 30 families containing 991 individuals using 25 microsatellite markers. The population consisting of 522 individuals, comprising eight full-sib families ranging from 45 to 107 progeny, was euthanized to determine the muscle fat content and record the growth-related traits. BW (g), TL (cm), and BT (mm) were measured in 522 progeny according to Part 3: measurement of characters of inspection of germplasm for cultured fishes reference standard (GB/T 18654.3-2008).

### Trait correlations and heritabilities

Preliminary statistical analysis of data were completed in SPSS 13.0, and variance-covariance components were calculated using ASReml 3.0 [46]. The genetic parameters of the three traits were estimated using a multiple-trait animal model:

$$ {\mathrm{Y}}_{\mathrm{i}\mathrm{j}} = {\upmu}_{\mathrm{i}} + {\mathrm{a}}_{\mathrm{i}\mathrm{j}} + {\mathrm{e}}_{\mathrm{i}\mathrm{j}} $$

where i represents the traits, Y_ij_ is the observation of trait i for animal j, μ_i_ is the mean value for trait i, а_ij_ is the random effect of trait i for animal j and e_ij_ is the random residual error for trait i for animal j.

Heritabilities (h^2^ = V_A_/V_P_) were calculated using ASReml single-trait analyses, where h^2^ is the estimated heritability, V_A_ is the additive genetic variance, and V_P_ the total phenotypic variance of the trait. Genetic correlations were calculated between the traits using ASReml bivariate analysis.

### Genotype data and linkage map

All individuals were genotyped using 250 microsatellite loci across regions of varying length on 50 LGs according to the common carp consensus linkage map [5]. The marker data and linkage map used in the study were described in Kuang et al. [7]. The genotype data of 233 markers, which fell into 50 common carp autosomes, were available after removal of monomorphic and unlink from a total of 250 markers. The average linkage map was used, which spanned a length of 3131.5 cM and the marker spacing per LG ranged from 6.1 to 25.8 cM with an average of 16.8 cM. Most of the LGs were moderately informative with an average information content range of 0.30–0.85. The average information content at the genome level was 0.63 [7].

### QTL mapping

The QTL analysis were performed using regression techniques [47] implemented by the online software package GridQTL [48]. GridQTL is a portlet environment (available at http://www.gridqtl.org.uk/) that permits the analysis of computationally intensive datasets.

The QTL analysis was carried out using two mapping strategies. First, the analysis was conducted using a sib-pair (SP) model, which used a variance method to analyze linkage, based on alleles that are identical-by-descent (IBD). Considering the full-sib pedigree information and the absence of genotypes on the parents, the default regression method for QTL linkage analysis was applied [49]. Across-family analyses were carried out for each trait and each LG to test for evidence of QTL segregation in all families for which information was available. A trait showing evidence for a single QTL was tested for the presence of two or more QTLs by fitting a two QTL model, which was followed by within-family analyses to identify specific families segregating for putative QTLs.

Second, the half-sib (HS) model for QTL-mapping analysis was carried out, which takes advantage of the disparity in female and male recombination rates (female:male ratio was 4.2:1) in common carp [5]. By using one parent at a time, we identified the parent that was segregating for the alleles defining the QTL. To test whether or not the QTLs were segregating in the eight full-sib families, a two-stage linear regression approach was applied separately for male and female parents [50]. The QTL mapping method was according to Houston et al. [51]; in their QTL detection of Atlantic salmon, there was a similar disparity between the male and female recombination ratio. Briefly, the detection of QTLs in the initial genome scan was founded on the sire-based analysis because the low recombination rate gives greater power to detect QTLs using low resolution with few markers per LG [52]. Dam-based analyses were subsequently conducted for the LGs that showed evidence of QTL segregation in the male map, because the larger female map had a greater resolving capacity to locate putative QTLs.

*F*-statistics were generated at 1-cM intervals along each LG to identify the most likely QTL position. Significance thresholds were determined by a permutation test [53], with 10,000 iterations performed to obtain a *P* < 0.05 and *P* < 0.01 chromosome-wide significance levels. The genome-wide thresholds were calculated by first obtaining a Bonferroni corrected *P-*value at the 0.05 and 0.01 significance level (given the 50 independent linkage groups in common carp, adjusted *P*-value = 0.05/50 and 0.01/50) and then obtaining the genome-wide *F*-ratio threshold at this adjusted *P*-value, using 10,000 permutations [50]. *F*-ratio values of *P* < 0.01 were considered significant QTLs, whereas those in which 0.01 < *P* < 0.05 were considered suggestive QTLs. The chromosomes that contained significant QTLs were tested for their significance at a genome-wide level of *P* < 0.05. The 95 % confidence intervals were estimated using bootstrap analyses with 10,000 iterations [54]. On each LG, regions were defined based on the occurrence of QTLs.

The percentage of the phenotypic variance explained (PVE) was calculated according to Knott et al.’s method [47]. In the sire- or dam-based analysis, the formula is:

$$ \mathrm{P}\mathrm{V}\mathrm{E} = 4\left[1\hbox{-} \left({\mathrm{MSE}}_{\mathrm{full}}/{\mathrm{MSE}}_{\mathrm{reduced}}\right)\right], $$

where MSE_full_ and MSE_reduced_ are the mean squared error of the full model and mean squared error of the reduced model (parameters fixed), respectively. Accordingly, the PVE was calculated from the combined sire- and dam-based analysis according to the formula:

$$ \mathrm{P}\mathrm{V}\mathrm{E} = 2\ \left(\left[1\hbox{-} {\left({\mathrm{MSE}}_{\mathrm{full}}/{\mathrm{MSE}}_{\mathrm{reduced}}\right)}^{\mathrm{Sire}}\right] + \left[1\hbox{-} {\left({\mathrm{MSE}}_{\mathrm{full}}/{\mathrm{MSE}}_{\mathrm{reduced}}\right)}^{\mathrm{Dam}}\right]\right). $$

Absolute t-value >2 for the test contrasting the effect of alternative alleles within each parent were used to identify putative heterozygous parents for the QTLs.

### Candidate genes

To identify candidate genes associated with growth-related traits, we took advantage of the currently available information from the common carp genome sequencing project [15], which is publicly available at the Common Carp Genome Base (www.carpbase.org).

## Availability of data and materials

All marker sequences have been deposited in the NCBI database, the GenBank accession number of the sequences were shown in Zhang et al. [5]. The linkage maps used in the study were described in Kuang et al. [7]. QTL data are available within the manuscript and its additional files.

## Ethics (and consent to participate)

This study was approved by the Animal Care and Use Committee of the Heilongjiang River Fisheries Research Institute at the Chinese Academy of Fishery Sciences.

## Additional file

Additional file 1: Table S1. Phenotypic correlations between growth-related traits in eight common. Table S2. List of suggestive (*P* < 0.05) and significant (*P* < 0.01) QTLs for body weight (BW) in eight common carp families based on half-sib analysis. Table S3. List of suggestive (*P* < 0.05) and significant (*P* < 0.01) QTLs for total length (TL) in eight common carp families based on half-sib analysis. Table S4. List of suggestive (*P* < 0.05) and significant (*P* < 0.01) QTLs for body thickness (BT) in eight common carp families based on half-sib analysis. (DOCX 123 kb)

## Abbreviations

BT: Body thickness
BW: Body weight
HS: Half-sib
IBD: Identical-by-descent
LG: Linkage group
MAS: Marker-assisted selection
NCBI: National Center for Biotechnology Information
PVE: Percentage of the phenotypic variance explained
QTL: Quantitative trait locus
SP: Sib-pair
TL: Total length
