# Supplementary material for: QTL variations for growth-related traits in eight distinct families of common carp (Cyprinus carpio)
Source: BMC Genet. 2016 May 5;17:65. doi: 10.1186/s12863-016-0370-9 (PMC4858896; doi:10.1186/s12863-016-0370-9)
Supplement: Additional file 1: Table S1. — Phenotypic correlations between growth-related traits in eight common. Table S2. List of suggestive (P < 0.05) and significant (P < 0.01) QTLs for body weight (BW) in eight common carp families based on half-sib analysis. Table S3. List of suggestive (P < 0.05) and significant (P < 0.01) QTLs for total length (TL) in eight common carp families based on half-sib analysis. Table S4. List of suggestive (P < 0.05) and significant (P < 0.01) QTLs for body thickness (BT) in eight common carp families based on half-sib analysis. (DOCX 123 kb) [file 12863_2016_370_MOESM1_ESM.docx]

Additional file 1

**Table S1.** Heritabilities and trait correlations among three growth-related traits estimated from multiple-trait animal models

| Trait | BW | TL | BT |
| --- | --- | --- | --- |
| BW | **0.23±0.13** | 0.92 | 0.88 |
| TL | 0.98 | **0.35±0.17** | 0.83 |
| BT | 0.98 | 0.93 | **0.25±0.13** |

BW, body weight; TL, total length; BT, body thickness; heritabilities are on the diagonal (in bold), phenotypic correlations on the right diagonal and genetic correlation on the left diagonal.

**Table S2.** List of suggestive (*P*< 0.05) and significant (*P* < 0.01) QTL for body weight in eight common carp families based on half-sib analysis

| **Family** | **Source** | **LG** | **Position**  **(cM)** | ***F*-**  **ratio** | ***F*-statistic threshold** | | | | **Estimate**  **(SE)** | **ABS**  **(t)** | **PVE** | **CI** | **Nearest marker** | **Related gene** |
| --- | --- | --- | --- | --- | --- | --- | --- | --- | --- | --- | --- | --- | --- | --- |
|  |  |  |  |  | **Chromosome-wide** | | **Genome-wide** | |  |  |  |  |  |  |
|  |  |  |  |  | **0.05** | **0.01** | **0.05** | **0.01** |  |  |  |  |  |  |
| F234 | Dam | 1 | 36 | 5.28 | 5.24 | 9.11 |  |  | 5.67(2.46) | 2.30 | 9.2 | 3-39 | HLJ2848 | pcdh2ac |
| F234 | Sire | 1 | 40 | 6.92 | 5.24 | 7.11 |  |  | 6.38(2.43) | 2.63 |  | 0-40 | HLJ2848 | pcdh2ac |
| F275 | Sire | 1 | 40 | 6.52 | 4.98 | 9.01 |  |  | 11.73(4.59) | 2.55 | 15.2 | 0-40 | HLJ2848 | pcdh2ac |
| F275 | Sire | 3 | 0 | 6.41 | 6.28 | 10.36 |  |  | -11.89(4.69) | 2.53 | 15 | 0-54 | HLJ3358 |  |
| F217 | Dam | 3 | 40 | 6.82 | 5.39 | 8.61 |  |  | 12.64(4.84) | 2.61 | 17 | 2-54 | HLJ2783 |  |
| F234 | Sire | 4 | 53 | 12.98 | 5.96 | 10.26 |  |  | -10.60(2.94) | 3.60 | 27.2 | 44-53 | HLJ2952 | mybcp2a |
| F234 | Dam | **4** | 53 | **22.40** | 5.39 | 8.89 | 15.81 | 18.11 | -13.49(2.85) | 4.73 |  | 44-53 | HLJ2952 | mybcp2a |
| F4039 | Sire | 6 | 89 | 7.82 | 5.99 | 9.19 |  |  | 12.15(4.34) | 2.80 | 18.2 | 38-136 | HLJ3356 |  |
| F259 | Dam | 6 | 117 | 6.52 | 6.44 | 10.92 |  |  | 20.67(8.09) | 2.55 | 20.8 | 0-136 | HLJ2994 | vps13b |
| F259 | Sire | 6 | 122 | 5.48 | 5.25 | 9.08 |  |  | 23.04(9.84) | 2.34 |  | 0-136 | HLJ2994 | vps13b |
| F4039 | Dam | 8 | 14 | 5.36 | 4.89 | 8.38 |  |  | -9.62(4.15) | 2.32 | 12 | 0-43 | HLJ3865 | apobl |
| F275 | Dam | 8 | 51 | 5.28 | 4.86 | 8.95 |  |  | -20.30(8.84) | 2.30 | 12 | 0-51 | HLJ3278 | stimla |
| F234 | Sire | 9 | 0 | 5.16 | 4.94 | 7.67 |  |  | -7.22(3.53) | 2.04 | 5.8 | 0-81 | HLJ3833 | farsb |
| F336 | Dam | 9 | 50 | 7.37 | 5.71 | 8.84 |  |  | -20.61(7.59) | 2.71 | 24.8 | 12-89 | CAFS1291 | msi2b |
| F217 | Dam | 10 | 4 | 8.92 | 5.53 | 9.38 |  |  | -18.07(6.05) | 2.99 | 22.4 | 3-41 | HLJ2593 |  |
| F373 | Sire | 11 | 5 | 14.13 | 6.33 | 10.25 |  |  | -11.57(3.07) | 3.76 | 42.2 | 0-49 | CAFS2305 |  |
| F234 | Dam | 13 | 0 | 8.38 | 5.63 | 9.04 |  |  | 7.16(2.47) | 2.89 | 13.2 | 0-62 | HLJ2650 |  |
| F259 | Sire | 14 | 46 | 6.72 | 6.65 | 10.2 |  |  | 14.42(5.56) | 2.59 | 24.4 | 0-51 | HLJ1887 |  |
| F373 | Dam | 16 | 7 | 8.87 | 5.49 | 9.96 |  |  | -13.30(4.46) | 2.98 | 27.6 | 0-27 | HLJ3114 | mbn12 |
| F275 | Sire | 17 | 13 | 8.6 | 6.72 | 11.21 |  |  | -12.46(4.25) | 2.93 | 20.4 | 0-71 | HLJ3852 |  |
| F234 | Dam | 18 | 51 | 6.51 | 5.27 | 7.44 |  |  | 7.77(3.04) | 2.55 | 10 | 14-60 | -- |  |
| F373 | Dam | 18 | 52 | 5.74 | 5.29 | 7.99 |  |  | 9.10(3.79) | 2.4 | 28 | 0-55 | HLJE299 |  |
| F373 | Sire | 18 | 60 | 12.64 | 5.32 | 8.14 |  |  | 11.32(3.18) | 3.55 |  | 36-60 | HLJE299 |  |
| F217 | Dam | 19 | 4 | 6.91 | 6.33 | 10.44 |  |  | 11.17(4.24) | 2.63 | 17.2 | 0-46 | CAFS1932 |  |
| F234 | Sire | 22 | 5 | 6.56 | 5.15 | 7.91 |  |  | -8.88(3.46) | 2.56 | 10 | 0-59 | HLJ2194 |  |
| F234 | Dam | 22 | 5 | 6.56 | 5.56 | 8.71 |  |  | -0.88(3.46) | 2.56 |  | 0-59 | HLJ2194 |  |
| F4039 | Sire | 24 | 34 | 10.85 | 3.92 | 6.55 |  |  | -13.12(3.98) | 3.29 | 20 | 12-56 | HLJ3754 |  |
| F4039 | Dam | 24 | 44 | 6.47 | 5.69 | 8.58 |  |  | 13.54(5.32) | 2.54 |  | 0-56 | HLJ3988 | casq2 |
| F259 | Sire | **24** | 44 | **40.00** | 5.57 | 8.99 | 16.37 | 23.26 | -25.42(4.02) | 6.32 | 48.7 | 23-56 | HLJ3988 | casq2 |
| F373 | Dam | 25 | 39 | 6.23 | 6.13 | 10.13 |  |  | -9.49(3.80) | 2.5 | 19.2 | 5-74 | HLJ3612 | lpcat1 |
| F217 | Sire | 25 | 67 | 6.72 | 6.48 | 10.93 |  |  | 10.45(4.03) | 2.59 | 16.6 | 41-74 | HLJ3969 |  |
| F4039 | Sire | 26 | 16 | 9.92 | 7.04 | 10.67 |  |  | 15.08(4.79) | 3.15 | 23.2 | 0-54 | HLJ2642 |  |
| F234 | Dam | 26 | 33 | 9.88 | 5.88 | 8.63 |  |  | 7.78(2.47) | 3.14 | 15.6 | 0-58.5 | CAFS2321 |  |
| F4039 | Dam | 27 | 12 | 7.09 | 6.37 | 10.31 |  |  | -11.05(4.15) | 2.67 | 16.4 | 0-70 | HLJ3272 |  |
| F336 | Dam | 30 | 19 | 7.7 | 7.05 | 11.65 |  |  | 14.15(5.10) | 2.78 | 26 | 0-117 | -- |  |
| F4039 | Dam | **30** | 22 | **14.35** | 6.54 | 10.13 | 14.31 | 18.63 | 23.37(6.17) | 3.79 | 35 | 6-123 | -- |  |
| F4039 | Sire | 30 | 111 | 6.50 | 6.07 | 9.53 |  |  | 14.93(5.85) | 2.55 |  | 0-127 | -- |  |
| F259 | Dam | 30 | 30 | 6.04 | 6.03 | 11.57 |  |  | 15.18(6.17) | 2.45 | 21.8 | 20-79 | CAFS1568 |  |
| F336 | Sire | 31 | 0 | 6.01 | 6.01 | 9.64 |  |  | -40.16(16.38) | 2.45 | 20 | 0-81 | HLJ2865 |  |
| F4039 | Dam | 31 | 7 | 6.30 | 5.71 | 9.14 |  |  | -11.39(4.53) | 2.51 | 14.4 | 0-81 | HLJ2190 | slain2 |
| F234 | Sire | 33 | 37 | 6.75 | 5.10 | 8.12 |  |  | -6.40(2.46) | 2.60 | 10.4 | 12-37 | HLJ1306 |  |
| F4039 | Dam | 37 | 16 | 6.10 | 5.27 | 8.59 |  |  | 10.22(4.14) | 2.47 | 14 | 0-21 | HLJ3542 |  |
| F275 | Dam | 38 | 46 | 6.43 | 5.26 | 8.49 |  |  | -10.28(4.06) | 2.54 | 15 | 0-46 | HLJ3291 |  |
| F336 | Dam | 39 | 0 | 5.75 | 5.69 | 8.96 |  |  | -9.62(4.01) | 2.4 | 19.2 | 0-29 | HLJ3573 |  |
| F336 | Dam | 41 | 15 | 9.23 | 5.35 | 9.32 |  |  | -13.75(4.50) | 3.05 | 31.2 | 0-24 | CAFS2332 | sept5b |
| F234 | Dam | 42 | 0 | 7.91 | 6.08 | 9.45 |  |  | -6.88(2.44) | 2.81 | 12.4 | 0-44 | CAFS1757 |  |
| F336 | Sire | 43 | 17 | 9.35 | 6.35 | 9.58 |  |  | 12.38(4.05) | 3.06 | 31.2 | 7-42 | HLJ360 |  |
| F275 | Dam | 45 | 48 | 11.02 | 6.99 | 12.32 |  |  | 13.48(4.06) | 3.32 | 26 | 35-131 | HLJ3597 |  |
| F217 | Sire | 45 | 121 | 6.55 | 6.07 | 9.74 |  |  | -10.69(4.53) | 2.36 | 13.4 | 13-131 | HLJ2129 | foxk2 |
| F373 | Sire | 45 | 120 | 6.71 | 6.69 | 11.14 |  |  | -10.55(4.07) | 2.59 | 20.8 | 43-131 | HLJ2129 | foxk2 |

LG, linkage group; Source indicates which parent segregated for the QTL; Position (cM) on the LG where the maximum F-statistic value was obtained; ABS(t), Absolute T value; PVE is the proportion of phenotypic variation explained by the QTL estimated using both the Sire and Dam analyses; CI, 95% confidence interval; LGs and F-ratios in bold are significant at genome-wide level.

**Table S3.** List of suggestive (*P*< 0.05) and significant (*P* < 0.01) QTL for total length in eight common carp families based on half-sib analysis

| **Family** | **Source** | **LG** | **Position**  **(cM)** | ***F*-**  **ratio** | ***F*-statistic threshold** | | | | **Estimate**  **(SE)** | **ABS**  **(t)** | **PVE** | **CI** | **Nearest marker** | **Related gene** |
| --- | --- | --- | --- | --- | --- | --- | --- | --- | --- | --- | --- | --- | --- | --- |
|  |  |  |  |  | **Chromosome-wide** | | **Genome-wide** | |  |  |  |  |  |  |
|  |  |  |  |  | **0.05** | **0.01** | **0.05** | **0.01** |  |  |  |  |  |  |
| F234 | Sire | 1 | 40 | 8.50 | 5.42 | 8.67 |  |  | 1.71(0.58) | 2.92 | 10.2 | 0-40 | HLJ3473 | ube2d2 |
| F234 | Dam | 1 | 37 | 5.84 | 5.61 | 8.64 |  |  | 1.32(0.60) | 2.20 |  | 3-38 | HLJ2848 |  |
| F259 | Dam | 1 | 34 | 10.79 | 6.31 | 9.79 |  |  | -4.86(1.47) | 3.29 | 37 | 6-32 | HLJ3473 | ube2d2 |
| F275 | Sire | 1 | 40 | 7.42 | 4.92 | 8.09 |  |  | 3.58(1.31) | 2.72 | 18 | 0-40 | HLJ2848 | pcdh2ac |
| F171 | Dam | 3 | 54 | 6.58 | 5.94 | 9.03 |  |  | -2.21(0.86) | 2.57 | 15.4 | 8-54 | HLJ2783 |  |
| F217 | Dam | 3 | 9 | 9.08 | 5.15 | 8.80 |  |  | 3.48(1.15) | 3.01 | 23.4 | 2-51 | HLJ3461 |  |
| F275 | Sire | 3 | 0 | 6.13 | 5.95 | 9.58 |  |  | -3.34(1.35) | 2.48 | 14.6 | 0-54 | HLJ2461 | clcn3 |
| F234 | Sire | 4 | 53 | 8.81 | 5.55 | 8.39 |  |  | -2.19(0.74) | 2.97 | 27 | 10-53 | HLJ2952 | mybcp2a |
| F234 | Dam | **4** | 50 | **20.54** | 5.48 | 8.63 | 14.43 | 18.44 | -3.33(0.73) | 4.53 |  | 41-53 | HLJ2952 | mybcp2a |
| F4039 | Sire | 6 | 90 | 10.31 | 6.32 | 10.55 |  |  | 3.91(1.21) | 3.21 | 24 | 63-112 | HLJ3356 | smg5 |
| F275 | Dam | 8 | 51 | 5.39 | 5.29 | 9.32 |  |  | -5.94(2.56) | 2.32 | 12.6 | 0-51 | HLJ3278 | stimla |
| F4039 | Dam | 8 | 12 | 9.34 | 5.11 | 8.14 |  |  | -3.48(1.14) | 3.06 | 21.8 | 0-41 | HLJ3865 | apobl |
| F234 | Sire | 9 | 0 | 5.23 | 5.09 | 9.62 |  |  | -1.99(0.87) | 2.29 | 7.8 | 0-77 | HLJ3833 | farsb |
| F336 | Dam | 9 | 54 | 7.49 | 5.34 | 10.04 |  |  | -4.97(1.81) | 2.74 | 25.2 | 18-89 | CAFS1291 | msi2b |
| F217 | Dam | 10 | 4 | 14.00 | 5.23 | 8.49 |  |  | -6.18(1.65) | 3.74 | 35.2 | 4-40 | HLJ2593 |  |
| F373 | Sire | 11 | 11 | 6.07 | 6.06 | 7.59 |  |  | -1.88(0.76) | 2.47 | 19.2 | 0-75 | HLJ3815 |  |
| F234 | Dam | 13 | 62 | 10.64 | 5.35 | 8.55 |  |  | 1.99(0.61) | 3.26 | 16.8 | 0-62 | HLJ2571 |  |
| F259 | Sire | 14 | 49 | 6.45 | 5.7 | 9.67 |  |  | 3.04(1.20) | 2.54 | 22.6 | 0-51 | HLJ1887 |  |
| F234 | Dam | 15 | 24 | 8.11 | 6.39 | 9.86 |  |  | -2.71(0.95) | 2.85 | 12.6 | 0-119 | -- |  |
| F373 | Dam | 16 | 25 | 7.99 | 5.04 | 8.32 |  |  | -2.33(0.82) | 2.83 | 25.4 | 1-40 | HLJ3559 |  |
| F275 | Sire | 17 | 10 | 7.80 | 6.59 | 10.65 |  |  | -3.42(1.22) | 2.79 | 19 | 0-72 | -- |  |
| F373 | Sire | 18 | 60 | 7.71 | 5.87 | 9.32 |  |  | 2.12(0.76) | 2.78 | 22.6 | 0-60 | HLJE299 |  |
| F373 | Dam | 18 | 52 | 6.50 | 6.01 | 9.47 |  |  | 2.23(0.87) | 2.55 |  | 0-55 | HLJE299 |  |
| F217 | Dam | 19 | 4 | 7.83 | 6.42 | 8.64 |  |  | -3.35(1.19) | 2.80 | 20.2 | 1-47 | CAFS1932 |  |
| F234 | Sire | 19 | 15 | 6.06 | 4.78 | 7.59 |  |  | -1.58(0.64) | 2.46 | 9.2 | 0-27 | HLJ456 | adiporla |
| F234 | Sire | 22 | 1 | 5.68 | 5.11 | 8.08 |  |  | -1.96(0.82) | 2.38 | 8.6 | 0-59 | HLJ2194 |  |
| F234 | Dam | 22 | 1 | 5.68 | 5.54 | 8.51 |  |  | -1.96(0.82) | 2.38 | 8.6 | 0-59 | HLJ2194 |  |
| F259 | Sire | **24** | 44 | **33.67** | 5.29 | 8.97 | 18.78 | 32.12 | -5.43(0.94) | 5.80 | 57.2 | 28-56 | HLJ3988 | casq2 |
| F259 | Dam | 24 | 55 | 8.08 | 6.31 | 11.32 |  |  | -3.23(1.13) | 2.84 |  | 0-56 | HLJE511 |  |
| F4039 | Sire | 24 | 34 | 7.08 | 4.92 | 7.64 |  |  | -3.04(1.14) | 2.66 | 16.4 | 3-56 | HLJ3754 |  |
| F217 | Sire | 25 | 65 | 6.46 | 6.00 | 8.32 |  |  | 2.72(1.16) | 2.34 | 13.6 | 38-74 | -- |  |
| F373 | Dam | 25 | 42 | 8.87 | 5.89 | 9.42 |  |  | -2.66(0.89) | 2.98 | 28.2 | 5-69.5 | HLJ3612 | lpcat1 |
| F234 | Dam | 26 | 31 | 9.01 | 6.45 | 9.90 |  |  | 1.88(0.62) | 3.00 | 14.2 | 0-86 | CAFS2321 |  |
| F259 | Dam | 30 | 29 | 8.66 | 7.05 | 10.34 |  |  | 3.96(1.34) | 2.94 | 30.2 | 20-78 | -- |  |
| F336 | Dam | 30 | 24 | 8.79 | 6.81 | 11.48 |  |  | 3.75(1.26) | 2.96 | 29.6 | 0-94 | -- |  |
| F4039 | Sire | 30 | 117 | 8.22 | 6.15 | 10.59 |  |  | 4.24(1.48) | 2.87 | 23.2 | 0-127 | -- |  |
| F4039 | Dam | 30 | 20 | 11.69 | 6.62 | 10.10 |  |  | 5.91(1.73) | 3.42 |  | 0-127 | -- |  |
| F336 | Sire | 31 | 0 | 6.76 | 6.16 | 10.14 |  |  | -9.77(4.07) | 2.40 | 19.2 | 0-81 | HLJ2865 |  |
| F4039 | Dam | 31 | 76 | 8.38 | 5.54 | 8.88 |  |  | -3.35(1.15) | 2.90 | 19.6 | 0-82 | HLJ3848 | med23 |
| F4039 | Dam | 33 | 9 | 5.85 | 5.79 | 9.40 |  |  | 3.50(1.44) | 2.42 | 13.4 | 2-37 | HLJ2143 |  |
| F275 | Dam | 38 | 46 | 8.74 | 5.37 | 7.62 |  |  | -3.38(1.14) | 2.96 | 21.2 | 0-46 | HLJ3291 |  |
| F171 | Sire | 39 | 3 | 6.24 | 4.39 | 7.97 |  |  | 3.49(1.39) | 2.50 | 14.4 | 0-3 | HLJ3573 |  |
| F336 | Dam | 39 | 0 | 7.62 | 5.28 | 10.22 |  |  | -2.69(0.97) | 2.76 | 25.6 | 0-29 | HLJ3573 |  |
| F336 | Dam | 41 | 14 | 11.56 | 5.73 | 9.71 |  |  | -3.62(1.06) | 3.40 | 38 | 0-22 | CAFS2332 | sept5b |
| F234 | Dam | 42 | 0 | 6.33 | 5.86 | 9.38 |  |  | -1.53(0.61) | 2.52 | 9.6 | 0-44 | CAFS1757 |  |
| F234 | Sire | 43 | 0 | 4.95 | 4.75 | 7.88 |  |  | 1.45(0.65) | 2.23 | 7.2 | 0-37 | HLJ3570 |  |
| F336 | Sire | 43 | 15 | 9.37 | 5.64 | 8.53 |  |  | 3.18(1.04) | 3.06 | 31.4 | 5-42 | HLJ360 |  |
| F275 | Dam | 45 | 50 | 11.75 | 7.16 | 10.58 |  |  | 4.13(1.21) | 3.43 | 28.4 | 33-131 | HLJ3597 |  |
| F336 | Sire | 45 | 51 | 8.81 | 6.88 | 10.31 |  |  | -3.54(1.19) | 2.97 | 29.6 | 9-131 | HLJ3597 |  |
| F373 | Sire | 45 | 121 | 5.97 | 5.35 | 9.47 |  |  | -2.31(0.94) | 2.44 | 18.8 | 19-131 | HLJ2129 | foxk2 |
| F4039 | Sire | 45 | 55 | 8.44 | 6.52 | 10.71 |  |  | -4.71(1.62) | 2.91 | 19.8 | 31-112 | HLJ3597 |  |

LG, linkage group; Source indicates which parent segregated for the QTL; Position (cM) on the LG where the maximum F-statistic value was obtained; ABS(t), Absolute T value; PVE is the proportion of phenotypic variation explained by the QTL estimated using both the Sire and Dam analyses; CI, 95% confidence interval; LGs and *F*-ratios in bold are significant at genome-wide level.

**Table S4.** List of suggestive (*P*< 0.05) and significant (*P* < 0.01) QTL for body thickness in eight common carp families based on half-sib analysis

| **Family** | **Source** | **LG** | **Position**  **(cM)** | ***F*-**  **ratio** | ***F*-statistic threshold** | | | | **Estimate**  **(SE)** | **ABS**  **(t)** | **PVE** | **CI** | **Nearest marker** | **Related gene** |
| --- | --- | --- | --- | --- | --- | --- | --- | --- | --- | --- | --- | --- | --- | --- |
|  |  |  |  |  | **Chromosome-wide** | | **Genome-wide** | |  |  |  |  |  |  |
|  |  |  |  |  | **0.05** | **0.01** | **0.05** | **0.01** |  |  |  |  |  |  |
| F234 | Sire | 1 | 38 | 5.06 | 5.06 | 8.46 |  |  | 0.36(0.16) | 2.25 | 9.8 | 0-40 | HLJ3942 |  |
| F234 | Dam | 1 | 36 | 7.94 | 5.66 | 8.81 |  |  | 0.45(0.16) | 2.82 |  | 7-40 | HLJ2848 | pcdh2ac |
| F259 | Dam | 1 | 33 | 6.75 | 5.36 | 8.06 |  |  | -1.20(0.46) | 2.60 | 23.6 | 6-34 | HLJ3473 | ube2d2 |
| F217 | Dam | 3 | 37 | 5.24 | 5.16 | 9.04 |  |  | 0.67(0.29) | 2.29 | 12.6 | 3-54 | -- |  |
| F234 | Sire | 3 | 8 | 5.26 | 4.79 | 7.33 |  |  | -0.43(0.18) | 2.29 | 7.8 | 0-54 | HLJ3358 |  |
| F259 | Sire | 3 | 54 | 6.13 | 5.06 | 8.59 |  |  | 0.85(0.34) | 2.48 | 21.4 | 1-54 | HLJ2783 |  |
| F234 | Dam | 4 | 52 | 14.33 | 5.56 | 9.31 |  |  | -0.75(0.19) | 3.78 | 22.4 | 40-53 | HLJ2952 | mybcp2a |
| F171 | Sire | 4 | 52 | 6.63 | 5.27 | 8.45 |  |  | -0.77(0.30) | 2.57 | 15.2 | 13-52 | HLJ2952 | mybcp2a |
| F4039 | Sire | 6 | 89 | 6.13 | 6.13 | 9.95 |  |  | 0.63(0.25) | 2.47 | 14 | 38-136 | HLJ3356 | smg5 |
| F336 | Dam | 9 | 48 | 9.24 | 5.46 | 8.06 |  |  | -1.51(0.49) | 3.04 | 31 | 13-89 | CAFS1291 | msi2b |
| F217 | Dam | 10 | 4 | 6.87 | 5.04 | 8.59 |  |  | -0.99(0.37) | 2.62 | 17 | 4-41 | HLJ2593 |  |
| F373 | Sire | 11 | 26 | 8.85 | 6.19 | 9.25 |  |  | -0.69(0.23) | 2.98 | 27.6 | 0-62 | CAFS975 |  |
| F234 | Dam | 13 | 0 | 8.82 | 5.67 | 7.57 |  |  | 0.48(0.16) | 2.97 | 13.8 | 0-62 | HLJ2650 |  |
| F373 | Sire | 13 | 0 | 10.66 | 6.51 | 10.64 |  |  | -3.51(1.07) | 3.27 | 33 | 0-60 | HLJ2650 |  |
| F259 | Sire | 14 | 49 | 6.36 | 6.35 | 10.21 |  |  | 0.92(0.36) | 2.52 | 22.2 | 0-51 | HLJ1887 |  |
| F234 | Sire | 16 | 19 | 5.14 | 5.13 | 8.44 |  |  | 0.38(0.17) | 2.27 | 7.6 | 0-52 | HLJ3559 |  |
| F373 | Dam | 16 | 7 | 5.25 | 5.13 | 9.08 |  |  | -0.77(0.33) | 2.29 | 16 | 0-53 | -- |  |
| F275 | Sire | 17 | 22 | 6.62 | 6.51 | 10.52 |  |  | -0.65(0.25) | 2.57 | 16 | 0-75 | HLJ3999 |  |
| F373 | Sire | 18 | 58 | 8.22 | 5.46 | 9.49 |  |  | 0.72(0.25) | 2.87 | 25.6 | 25-60 | HLJE299 |  |
| F217 | Sire | 19 | 4 | 5.29 | 4.73 | 7.80 |  |  | 0.62(0.27) | 2.30 | 12.4 | 0-50 | CAFS2227 |  |
| F217 | Dam | 19 | 4 | 5.12 | 4.86 | 9.52 |  |  | 0.60(0.26) | 2.26 |  | 1-47 | CAFS2227 |  |
| F234 | Sire | 19 | 26 | 4.39 | 4.18 | 8.51 |  |  | -0.34(0.16) | 2.09 | 6.2 | 0-42 | CAFS1317 |  |
| F234 | Sire | 22 | 17 | 9.52 | 4.93 | 7.67 |  |  | -0.73(0.23) | 3.09 | 14.8 | 0-59 | HLJ2491 | ical1 |
| F234 | Dam | 22 | 17 | 9.52 | 5.03 | 8.38 |  |  | -0.73(0.23) | 3.09 |  | 0-58 | HLJ2491 | ical1 |
| F234 | Dam | 24 | 56 | 6.49 | 5.82 | 8.27 |  |  | -0.41(0.16) | 2.55 | 9.8 | 6-56 | HLJE511 |  |
| F259 | Sire | **24** | 40 | **28.06** | 5.72 | 9.75 | 18.91 | 25.98 | -1.56(0.29) | 5.29 | 38.6 | 24-56 | HLJ3988 | casq2 |
| F4039 | Sire | 24 | 56 | 14.01 | 4.47 | 7.96 |  |  | -0.85(0.22) | 3.74 | 32.2 | 2-56 | HLJE511 |  |
| F217 | Sire | 25 | 67 | 6.86 | 5.66 | 10.02 |  |  | 0.65(0.24) | 2.62 | 17 | 42-74 | -- |  |
| F259 | Sire | 25 | 74 | 4.64 | 4.51 | 8.07 |  |  | -1.13(0.52) | 2.15 | 15.6 | 0-74 | HLJ3952 |  |
| F234 | Dam | 26 | 28 | 6.70 | 6.64 | 10.24 |  |  | 0.45(0.17) | 2.58 | 10.2 | 0-86 | CAFS2321 |  |
| F4039 | Sire | 26 | 11 | 7.79 | 6.75 | 9.52 |  |  | 0.67(0.23) | 2.79 | 18.2 | 0-64 | HLJ2642 |  |
| F259 | Dam | 30 | 31 | 6.82 | 6.81 | 10.29 |  |  | 1.04(0.40) | 2.61 | 23.8 | 7-77 | -- |  |
| F336 | Dam | 30 | 20 | 6.45 | 6.42 | 10.57 |  |  | 0.79(0.31) | 2.54 | 21.6 | 0-113 | HLJ2170 |  |
| F4039 | Dam | 30 | 22 | 15.26 | 6.24 | 10.28 |  |  | 1.38(0.35) | 3.91 | 34.6 | 7-127 | -- |  |
| F336 | Sire | 31 | 55 | 6.25 | 6.23 | 9.49 |  |  | -0.92(0.36) | 2.50 | 20.8 | 0-78 | HLJ3471 |  |
| F234 | Sire | 33 | 37 | 5.90 | 5.09 | 7.97 |  |  | -0.40(0.16) | 2.43 | 8.8 | 4-37 | HLJ1306 |  |
| F336 | Dam | 39 | 0 | 6.77 | 5.96 | 9.55 |  |  | -0.68(0.26) | 2.61 | 22.8 | 0-29 | HLJ3573 |  |
| F336 | Dam | 41 | 16 | 11.31 | 5.91 | 9.07 |  |  | -0.99(0.29) | 3.36 | 37.2 | 0-24 | CAFS2332 | sept5b |
| F234 | Dam | 42 | 0 | 5.50 | 5.41 | 10.11 |  |  | -0.38(0.16) | 2.35 | 8.2 | 0-45 | CAFS1757 |  |
| F275 | Dam | 42 | 9 | 5.71 | 5.08 | 9.08 |  |  | -1.12(0.47) | 2.39 | 13.6 | 0-45 | -- |  |
| F336 | Sire | 43 | 18 | 5.59 | 5.44 | 8.48 |  |  | 0.64(0.27) | 2.36 | 18.6 | 12-42 | HLJ360 |  |
| F217 | Sire | 45 | 58 | 5.54 | 5.43 | 10.09 |  |  | -0.69(0.29) | 2.35 | 13.4 | 6-131 | -- |  |
| F275 | Dam | 45 | 131 | 9.89 | 6.62 | 10.92 |  |  | 0.78(0.24) | 3.14 | 10.2 | 0-131 | HLJ2129 | foxk2 |

LG, linkage group; Source indicates which parent segregated for the QTL; Position (cM) on the LG where the maximum F-statistic value was obtained; ABS(t), Absolute T value; PVE is the proportion of phenotypic variation explained by the QTL estimated using both the Sire and Dam analyses; CI, 95% confidence interval; LGs and *F*-ratios in bold are significant at genome-wide level.
